# Long Noncoding RNA BCYRN1 Recruits BATF to Promote TM4SF1 Upregulation and Enhance HCC Cell Proliferation and Invasion

**DOI:** 10.1155/2022/1561607

**Published:** 2022-06-11

**Authors:** Ya Liu, Meng-jie Liu, Min Jiao, Li-li Jiang, Xiao Fu, Wen-juan Wang

**Affiliations:** ^1^The First Affiliated Hospital of Xi'an Jiaotong University, Xi'an, Shaanxi 710061, China; ^2^Department of Medical Oncology, The First Affiliated Hospital of Xi'an Jiaotong University, Xi'an, Shaanxi 710061, China

## Abstract

Hepatocellular carcinoma (HCC) is a common form of cancer for which a subset of reliable clinical biomarkers has been defined. However, other factors including long noncoding RNAs (lncRNAs) can also regulate HCC development. This study was thus designed to understand how the lncRNA Brain cytoplasmic RNA 1 (BCYRN1) modulates HCC progression. Bioinformatics approaches were used to identify genes, lncRNAs, and transcription factors that were differentially expressed in the context of HCC, after which the relative expression of BCYRN1 in HCC and control tissues was assessed via qPCR. The ability of BCYRN1 to bind the transcription factor BATF was further evaluated in an RNA immunoprecipitation (RIP) assay, while chromatin immunoprecipitation (ChIP) was used to gauge the binding of the TM4SF1 promoter by BATF. Luciferase reporter assays were also used to assess the association between BCYRN1 and the TM4SF1 promoter. Subsequent loss- and gain-of-function assays were then conducted to explore the effects of altering BCYRN1 expression levels on the proliferative, invasive, and migratory activity of HCC cells. BCYRN1 upregulation was associated with poorer clinical outcomes in HCC patients, and knocking down this lncRNA impaired HCC cell migration and invasion. From a mechanistic perspective, BATF was recruited to the TM4SF1 promoter by BCYRN1, and reducing the expression of this lncRNA was sufficient to constrain xenograft tumor growth in mice. These results highlight BCYRN1 as a putative therapeutic target in HCC tumors.

## 1. Introduction

Hepatocellular carcinoma (HCC) is the most prevalent form of primary liver cancer and the third leading cause of cancer-related death worldwide [1]. HCC patients often exhibit a poor prognosis associated with high rates of tumor metastasis and recurrence, with few effective treatments being available given that the disease is often only diagnosed when in an advanced stage [2]. The mechanistic basis for HCC onset and progression remains to be fully clarified, and such clarification has the potential to guide the design of more efficacious treatments capable of improving patient outcomes.

Long noncoding RNAs (lncRNAs) lack the ability to encode proteins despite being over 200 nucleotides long [3]. However, lncRNA dysregulation is a common cancer hallmark [4], and these transcripts can modulate cellular biology by controlling protein-protein or protein-DNA interactions through scaffold-like activity, in addition to potentially functioning as competing endogenous RNAs that sequester miRNAs in a sequence-specific fashion [5–7]. The glutaminase antisense lncRNA (GLS-AS), for example, can interact with the GLS pre-mRNA to posttranscriptionally suppress its expression in pancreatic tumors [8]. Further research is necessary to clarify the roles of most lncRNAs in HCC.

In one recent report, the downregulation of LINC01093 was found to be associated with more advanced disease stage and poorer overall survival (OS) in individuals with HCC (9), while the overexpression of this lncRNA disrupted the malignant activities of HCC cells via interacting with IGF2BP1 and GLI [9]. Bioinformatics studies have further indicated that decreased CTC-297N7.9 levels are associated with HCC malignancy [10], underscoring the ability of these abovementioned lncRNAs to suppress HCC tumor growth. Altered TM4SF1 expression has previously been detected and linked to enhanced HCC cell invasion [11]. BATF is an oncogenic transcription factor that is thought to mediate HCC development, indicating that it may be a valuable therapeutic target in the context of HCC [12]. However, there have been few studies exploring the mechanistic role of the lncRNA brain cytoplasmic RNA 1 (BCYRN1) in HCC. Therefore, our present study primarily focused on the BCYRN1 regulatory network in HCC. As such, in the present report, we explore the associations between the lncRNA BCYRN1, BATF, and TM4SF1 in HCC in an effort to elucidate novel treatment approaches for this deadly cancer.

## 2. Materials and Methods

### 2.1. Sample Collection

In total, samples were collected from 100 HCC patients. The Ethics Committee of the First Affiliated Hospital, College of Medicine of Xi'an Jiaotong University, approved this study, with patients having provided written informed consent. Patients eligible for inclusion were those with pathologically confirmed HCC who had not undergone preoperative chemotherapy or radiotherapy. Patients were excluded if they had a history of autoimmunity, were suffering from acute or chronic infectious diseases, or exhibited severe impairment of the lungs, liver, or heart. Patient overall survival (OS) was defined as the time between treatment and death.

### 2.2. Cell Culture and Transfection

HCCLM3 cells were purchased from the American Type Culture Collection (ATCC) and grown in DMEM containing 10% FBS and penicillin/streptomycin (Invitrogen) in a 5% CO_2_ 37°C incubator. Cells were passaged when 80% confluent.

For appropriate assays, cells were transfected with in-house prepared plasmids including a negative control overexpression vector (OV-NC) + a negative control shRNA (sh-NC), OV-BCYRN1 + sh-NC, OV-NC + sh-BCYRN1, OV-NC, OV-BCYRN1, sh-NC, sh-BCYRN1, OV-BCYRN1 + sh-BATF, OV-BCYRN1 + sh-TM4SF1, OV-BCYRN1 + OV-BATF + sh-NC, and OV-BCYRN1 + OV-BATF + sh-TM4SF1. For transfection, cells were added to 6-well plates until 70-80% confluent and were transfected using Lipofectamine 3000 (Cat. L3000008, Thermo Fisher Scientific). At 8 h posttransfection, media were replaced, and cells were cultured for 48 h prior to downstream experimental use.

### 2.3. Quantitative Real-Time PCR (qPCR)

TRIzol (Cat. 15596026, Invitrogen) was used to isolate RNA from samples, after which a NanoDrop ND-1000 instrument (Thermo Scientific, DE, USA) was used to quantify RNA levels in isolated samples. A reverse transcription kit (RR047A, Takara) was then used to prepare cDNA, and qPCR was then performed with a SYBR Premix EX Taq kit (RR420A, Takara) and an ABI 7500 qPCR instrument. The 2^−*ΔΔ*Ct^ method was used to assess relative gene expression in triplicate experiments, with GAPDH as a normalization control.

### 2.4. Colony Formation Assay

Treated HCC cells were added to 6-well plates for 2 weeks to facilitate colony formation, after which crystal violet staining (2%) was performed and colonies were enumerated with an inverted microscope (Olympus, Tokyo, Japan). Assays were conducted in triplicate.

### 2.5. Fluorescence In Situ Hybridization (FISH)

BCYRN1 localization within cells was assessed via FISH using a Ribo™ lncRNA FISH probe Mix (Red) (Guangzhou RiboBio Co., Ltd.). Briefly, cells were added to coverslips in 24-well plates and were cultured overnight to 80% confluence, at which time they were fixed using 4% paraformaldehyde (1 mL) and treated with protease K, glycine, and ethyl phthalate (2 *μ*g/mL). Cells were then treated for 1 h with 250 *μ*L of a prehybridization solution at 42°C, followed by an additional 1 h incubation in hybridization buffer (250 *μ*L) supplemented with the probe (300 ng/mL) at 42°C. DAPI (1 : 1000 in PBST) was then applied for 5 min to counterstain nuclei, and cells were mounted with an antifluorescence quencher and imaged via fluorescence microscope (Olympus Optical Co., Ltd), with five random fields of view per sample being examined.

### 2.6. RNA Immunoprecipitation (RIP) Assay

A RIP kit (Cat. MAGNARIP02, Millipore Co., Ltd) was used to assess interactions between BATF and BCYRN1. Briefly, cells were lysed for 5 min in RIP assay buffer, followed by centrifugation for 10 min at 12,000 × g at 4°C. Supernatants were then collected and combined for 5 min with control IgG (ab172730, 1 : 100, Abcam) or anti-BATF-coated magnetic beads (2 *μ*g per 1 mL of cell lysate) overnight at 4°C. Bead-protein complexes were then washed in 1000 *μ*L of RIP wash buffer, treated with protease K, and RNA was isolated for PCR analysis.

### 2.7. Western Blotting

RIPA buffer (P0013B, Beyotime Biotechnology Co., China) containing PMSF was used to extract protein from samples of interest, after which a BCA assay (Cat. 23225, Thermo Fisher Scientific, Waltham, MA, USA) was confirmed to assess protein concentrations. Samples (50 *μ*g) were then boiled for 5 min in 2× SDS loading buffer (P0015B, Beyotime Biotechnology Co., China), followed by SDS-PAGE separation, and transferred to PVDF membranes. Blots were blocked for 1 h with 5% nonfat milk followed by overnight incubation with mouse monoclonal anti-E-cadherin (1 : 1000, ab76055, Abcam, Cambridge, MA, USA), rabbit polyclonal anti-MMP2 (1 : 1000, ab97779, Abcam, Cambridge, MA, USA), rabbit monoclonal anti-BATF (1 : 1000, #8638, Cell Signaling Technology, Danvers, MA), rat anti-TM4SF1 (1 : 2000, ab113504, Abcam, Cambridge, MA, USA), or rabbit polyclonal anti-GAPDH (1 : 1000, ab9485, Abcam, Cambridge, MA, USA) at 4°C. Blots were then probed with HRP-linked goat-anti-rabbit IgG (1 : 20,000, ab205718, Abcam, Cambridge, MA, USA) or anti-mouse IgG (1 : 20,000, ab6789, Abcam, Cambridge, MA, USA) for 1 h, followed by the use of an enhanced chemiluminescence (ECL) kit (BB-3501, Ameshame, united kingdom) for protein band detection, with a gel imaging instrument (ChemiDoc XRS+, Bio-Rad, Hercules, CA, USA) being used for imaging. Densitometric analyses were conducted with the Quantity One v4.6.2 software, and GAPDH was used as a reference control.

### 2.8. Immunohistochemistry (IHC)

Human liver tumors were harvested, immediately fixed in formalin, and embedded in paraffin according to the standard protocol. The tissue samples were cut to 5 *μ*m thickness using a cryotome. For immunohistochemistry, the sections were boiled in antigen retrieval solution over 20 min to expose antigens. Slides were blocked in 10% normal goat serum (Beyotime Biotechnology Co., China) in PBS for 1 hour at room temperature. Then, the tissues were incubated with primary antibody (TM4SF1 rabbit polyclonal antibody, 1 : 50 dilution, ab113504, Abcam, USA) at 4°C for 90 min, followed by incubation with a secondary antibody (goat-anti-rabbit IgG, 1 : 50 dilution, Abcam, USA) for 1 h at room temperature. Cells were then incubated with strep-avidin-biotin complex (SABC) at 37°C for 30 min and DAB (3,3′-diaminobenzidine) was used for color development. After each incubation, sections were washed three times with Tris Buffered Saline (TBS) for 10 s per wash. A hematoxylin staining kit (Beyotime Biotechnology Co., China) was then used for counterstaining, and photomicrographs were taken on a Leica microscope equipped with a CCD camera.

### 2.9. Luciferase Reporter Assay

The JASPAR tool was used to predict BATF binding sites in the TM4SF1 promoter. HEK293T cells (Type Culture Collection of the Chinese Academy of Sciences, Shanghai) were transfected with WT or mutant (MUT) TM4SF1 luciferase reporter vectors and the corresponding vectors, and at 48 h posttransfection, lysates were analyzed with a Firefly Luciferase Reporter Gene Assay Kit (Cat. E1910, Promega, USA) using a microplate reader (MK3, Thermo Fisher Scientific) at 560 nm.

### 2.10. Chromatin Immunoprecipitation (ChIP)

Cells were first fixed for 10 min with formaldehyde, after which chromatin was fragmented via ultrasonication (15 cycles; 10 s/cycle with 10 s between cycles). Samples were then spun down for 10 min at 12,000 × g at 4°C, and supernatants were collected and separated into tubes containing either control IgG (ab172730, 1 : 100, Abcam) or anti-BATF (2 *μ*g of cell lysate) followed by incubation overnight at 4°C. Protein agarose/sepharose was then used to precipitate DNA protein complexes, and samples were spun for 5 min at 12,000 × g at 4°C. Cross-linking was then reversed at 65°C overnight, after which phenol/chloroform was used to recover and purify DNA fragments, and BATF binding to the TM4SF1 promoter was assessed with appropriate primers (Table S1).

### 2.11. Transwell Assays

Tumor cells were resuspended in serum-free RPMI-1640 in the upper chamber of a 24-well Transwell insert (Millipore Corp., Bedford, MA, USA) to test HCC cell migratory activity, with media containing 10% FBS being added to the lower chamber. Following 48 h incubation, cells in the upper chamber were removed, and those that had migrated to the lower chamber were fixed, stained using DAPI, and counted. A Cell Invasion Assay Kit (ECM550, Millipore Corp., Bedford, MA, USA) was used based on provided directions to analyze tumor cell migration, with cells in five randomly selected fields of view being counted for analyses.

### 2.12. Murine Xenograft Tumor Models

Female nude BALB/c mice (18-25 g, 4 weeks old, *n* = 35) were housed under specific pathogen-free conditions. HCC cells transfected with sh-NC, sh-BCYRN1, OV-NC + sh-NC, OV-BCYRN1 + OV-BATF + sh-NC, or OV-BCYRN1 + OV-BATF + sh-TM4SF1A (2 × 10^6^) suspended in a 100 *μ*L mixture of normal saline and Matrigel (1 : 1) were subcutaneously implanted in these mice, and tumor volume (*V*, in mm^3^) was measured based upon tumor length (*A*) and width (*B*) using the following formula: *V* = (*A* × *B*^2^)/2. Tumor volumes were monitored over time for 24 d, after which mice were euthanized and tumors were collected and imaged. The Laboratory Animal Care and Use Committee of our Hospital approved all animal studies.

### 2.13. Statistical Analysis

SPSS v18.0 (IBM Corp., NY, USA) was used for all statistical testing. Data are means ± SD. The Kaplan-Meier curves and log-rank tests were used to assess patient survival. *P* < 0.05 was the significance threshold for this study.

## 3. Results

### 3.1. The Upregulation of BCYRN1 in HCC Patient Tumors Is Associated with Poor Patient Outcomes

**Figure 1:**
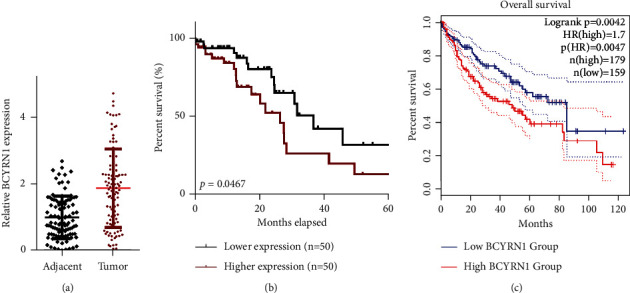
BCYRN1 upregulation is associated with poorer HCC patient outcomes. (a) qPCR was used to assess BCYRN1 expression in HCC tumor (*n* = 100) and paracancerous liver tissues (*n* = 100), ^∗^*P* < 0.01 vs. paracancerous tissues. (b) The relationship between BCYRN1 expression and HCC patient prognosis was examined via the Kaplan-Meier approach. (c) The relationship between BCYRN1 expression and HCC patient prognosis was examined through a GEPIA analysis (*P* < 0.035). Data are means ± standard deviation (SD) and were compared via paired *t*-tests unless otherwise indicated.

To explore the functional implications of differential BCYRN1 expression in HCC, we analyzed the levels of this lncRNA in 100 archived HCC patient samples via qPCR, revealing it to be significantly overexpressed in tumors relative to paracancerous tissues (Figure 1(a)). Notably, the OS of patients expressing high BCYRN1 levels was significantly reduced relative to that of patients expressing low levels of BCYRN1 (Figure 1(b)), and analyses of the Kaplan-Meier plotter data conducted using the GEPIA server demonstrated that increased BCYRN1 expression was indeed linked to poorer HCC patient OS (Figure 1(c)). These results thus suggested that BCYRN1 is dysregulated in HCC in a manner correlated with patient survival.

### 3.2. BCYRN1 Knockdown Suppresses HCC Cell Malignancy and Induces EMT

**Figure 2:**
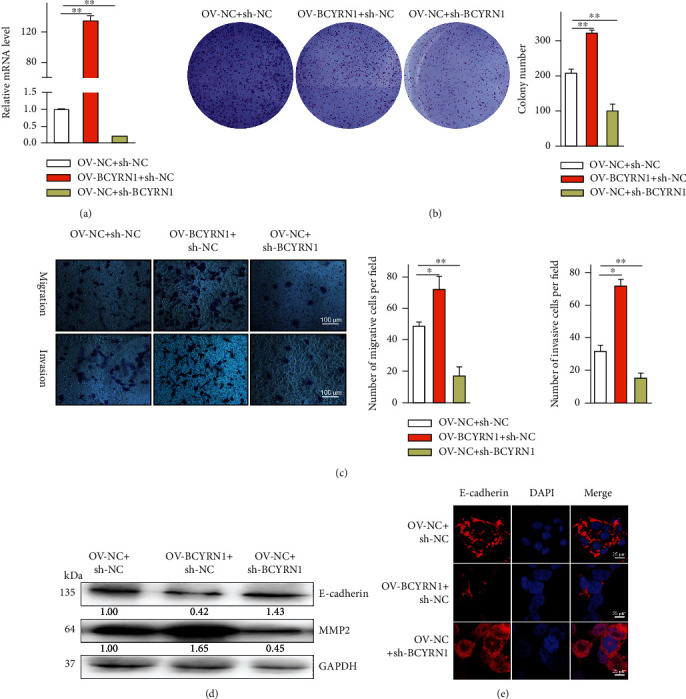
BCYRN1 silencing disrupts the migratory and invasive activity of HCC cells while promoting EMT induction. (a) The impact of BCYRN1 knockdown or overexpression in HCCLM3 cells was confirmed via qPCR. (b) The proliferation of cells transfected with OV-BCYRN1 or sh-BCYRN1 was established via colony formation assay. (c) HCCLM3 cell migration and invasion were evaluated in a Transwell assay (×200). (d) E-cadherin and MMP2 levels in HCCLM3 cells were evaluated via Western blotting, with GAPDH as a loading control. (e) Immunofluorescent staining for E-cadherin was assessed following OV-BCYRN1 or sh-BCYRN1 transfection. Data are means ± SD from triplicate experiments. ^∗^*P* < 0.05.

Gain- and loss-of-function assays in HCCLM3 cells were next conducted to explore the functional effects of BCYRN1 expression in HCC by overexpressing or knocking down this lncRNA (Figure 2(a)). The proliferation of HCC cells in a colony formation assay was markedly enhanced by BCYRN1 overexpression, whereas knocking it down had the opposite effect (Figure 2(b)). Similarly, BCYRN1 overexpression enhanced HCCLM3 cell invasion and migration in a Transwell assay (Figure 2(c)), while BCYRN1 knockdown resulted in the impairment of such malignant activity. Western blotting was then used to assess the expression of the invasion marker MMP and the epithelial marker E-cadherin (Figure 2(d)), revealing that OV-BCYRN1 + sh-NC treatment reduced E-cadherin expression and augmented MMP2 expression relative to OV-NC + sh-NC treatment, whereas OV-NC + sh-BCYRN1 had the opposite effect. Immunofluorescent staining for E-cadherin in these cells yielded comparable findings (Figure 2(e)). Together, these results suggested that knocking down BCYRN1 can inhibit the HCC cell malignancy.

### 3.3. BCYRN1 Upregulates TM4SF1 by Recruiting the BATF Transcription Factor

**Figure 3:**
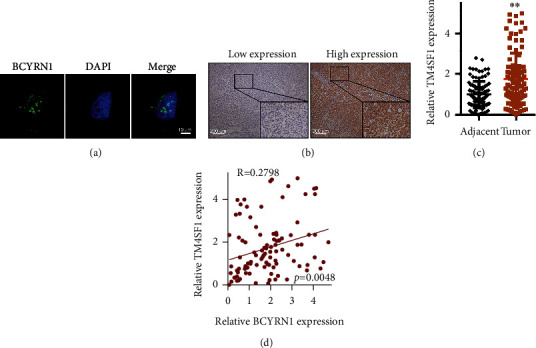
BCYRN1 expression is correlated with that of TM4SF1. (a) BCYRN1 localization within HCCLM3 cells was assessed via FISH assay (×630). (b) IHC was used to gauge TM4SF1 expression in HCC. (c) qPCR was used to assess TM4SF1 expression in HCC tissues, with GAPDH for normalization. (d) Correlations between BCYRN1 and TM4SF1 expression in HCC tissues were evaluated via Spearman's analyses (*n* = 100). ^∗∗^*P* < 0.01.

FISH assays revealed BCYRN1 to be present in the cytoplasm and nucleus of HCCLM3 cells (Figure 3(a)), indicating that this lncRNA had the potential to modulate gene expression in both of these compartments. The lncMAP database predicted BCYRN1 to be capable of regulating TM4SF1 expression indirectly via interacting with the transcription factor BATF (Fig. S1A) (13). BATF expression in the UALCAN database was therefore examined (Fig. S1B and 1C), as was TM4SF1 expression (Fig. S1D and 1E). TM4SF1 expression was found to be markedly enhanced in tumor tissues relative to normal controls via immunohistochemistry (Figure 3(b)), and this was confirmed via qPCR (Figure 3(c)). Notably, a positive correlation between BCYRN1 and TM4SF1 expression was observed in HCC tissues (Figure 3(e)).

**Figure 4:**
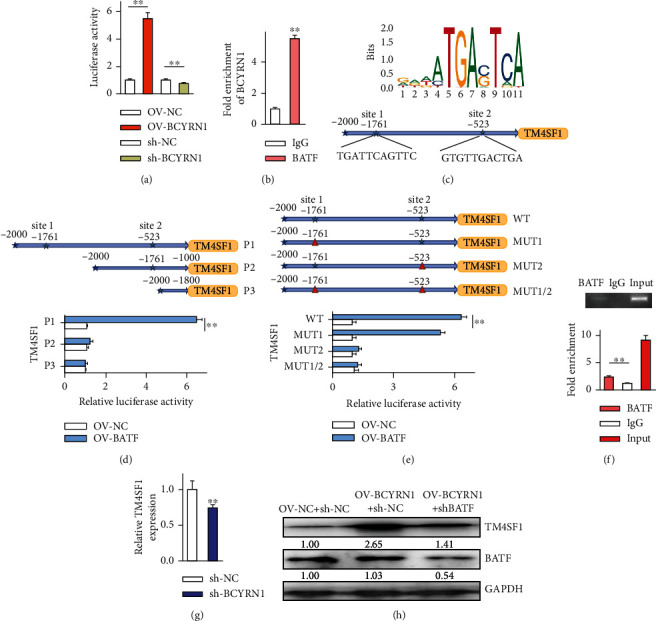
BCYRN1 recruits BATF to drive TM4SF1 expression. (a) The impact of BCYRN1 on TM4SF1 promoter activity was examined via the luciferase reporter assay. (b) Interactions between BATF and BCYRN1 were confirmed through a RIP assay, ^∗∗^*P* < 0.01 vs. IgG. (c) Putative BATF binding sites within the TM4SF1 promoter. (d) HCCLM3 cells were transfected with BATF expression vectors and truncated TM4SF1 luciferase reporter constructs or (e) mutant TM4SF1 luciferase reporter constructs in a luciferase reporter assay, ^∗∗^*P* < 0.01 vs. the OV-NC group. (f) BATF binding to TM4SF1 promoter site 2 was assessed via ChIP assay, ^∗∗^*P* < 0.01 vs. IgG. (g) BATF-mediated upregulation of TM4SF1 by BATF following BCYRN1 silencing within HCCLM3 cells. (h) TM4SF1 and BATF levels were assessed by Western blotting, with GAPDH for normalization. Data are means ± SD from triplicate experiments and were compared via paired *t*-tests when normally distributed.

A luciferase reporter assay was next conducted, revealing that BCYRN1 overexpression significantly enhanced TM4SF1 promoter activation relative to OVV-NC transfection (*P* < 0.05), whereas the knockdown of this lncRNA had the opposite effect, indicating that BCYRN1 can regulate TM4SF1 expression (Figure 4(a)). To further understand how BCYRN1 regulates TM4SF1, a RIP assay was performed to assess BCYRN1 binding to BATF (Figure 4(b)). This analysis confirmed that BATF was bound by this lncRNA as evidenced by increased BCYRN1 levels in samples precipitated with anti-BATF relative to those prepared using control IgG. To detect specific BATF protein binding sites in the TM4SF1 promoter region, the JASPAR tool was used, revealing two such putative binding sites (Figure 4(c)). A subsequent luciferase reporter assay confirmed that BATF was able to bind to site 2 (-523) within the TM4SF1 promoter (Figures 4(d) and 4(e)). A ChIP assay was then conducted to confirm this binding activity in HCCLM3 cells (Figure 4(f)), revealing a significant increase in amplification product levels when using site 2 primers relative to distal primers in samples precipitated with anti-BATF relative to those prepared with control IgG, confirming that BATF preferentially binds to this site 2 region (GTGTTGACTGA). We then knocked down BCYRN1 in HCCLM3 cells and repeated this ChIP assay (Figure 4(g)), revealing that BCYRN1 knockdown ablated the previously observed enrichment of TM4SF1 site 2 amplification products following BATF immunoprecipitation, confirming the direct regulatory relationship between these molecules.

To further validate that BCYRN1 regulates TM4SF1 expression via BATF binding, cells were next treated with OV-NC + sh-NC, OV-BCYRN1 + sh-NC, or OV-BCYRN1 + sh-BATF. TM4SF1 levels were significantly elevated in cells treated with OV-BCYRN1 + sh-NC relative to those treated with control vectors, whereas BATF levels were unaffected, while both BATF and TM4SF1 levels were decreased in cells treated with OV-BCYRN1 + sh-BATF relative to cells treated with OV-BCYRN1 + sh-NC (Figure 4(h)), suggesting that BCYRN1 regulates TM4SF1 expression via recruiting BATF.

### 3.4. BCYRN1 Promotes HCC Cell Malignancy by Recruiting BATF to Thereby Upregulate TM4SF1

**Figure 5:**
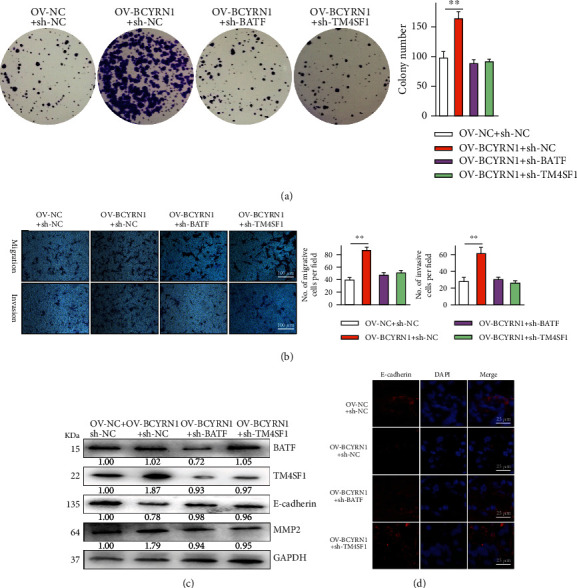
BCYRN1 overexpression enhances BATF-mediated TM4SF1 upregulation to modulate HCC proliferation, migration, and invasion. (a) Proliferation was examined via colony formation assay. (b) HCC cell migration and invasion were evaluated in a Transwell assay (×200). (c) BATF, TM4SF1, E-cadherin, and MMP2 levels in HCC cells were assessed by Western blotting, with GAPDH for normalization. (d) Immunofluorescent staining of E-cadherin levels. Data are means ± SD from triplicate experiments.

To understand how BCYRN1 impacts HCC cell characteristics, HCCLM3 cells were next transfected with OV-NC + sh-NC, OV-BCYRN1 + sh-NC, OV-BCYRN1 + sh-BATF, or OV-BCYRN1 + sh-TM4SF1, and their proliferative and invasive activities were then assessed. While BCYRN1 overexpression enhanced the proliferation of these cells in a colony formation assay (Figure 5(a)), this was reversed when BATF or TM4SF1 was simultaneously knocked down. Similarly, the knockdown of BATF or TM4SF1 was sufficient to reverse BCYRN1 overexpression-mediated enhancement of HCC cell migration and invasion (Figure 5(b)). Western blotting indicated that OV-BCYRN1 transfection reduced E-cadherin expression and increased SNAIL and TM4SF1 protein levels in HCC cells (Figure 5(c)), while sh-BATF or sh-TM4SF1 cotransfection reverses these effects. E-cadherin immunofluorescent staining assays yielded comparable results (Figure 5(d)). BATF silencing significantly decreased BATF expression, which was unaffected by TM4SF1 knockdown. When BATF and BCYRN1 were both overexpressed, this further enhanced HCC migration, proliferation, and invasion in a manner that was reversed by TM4SF1 silencing (Fig. S2). BCYRN1 thus enhances HCC cell malignancy at least in part through a mechanism dependent upon the recruitment of BATF to promote TM4SF1 upregulation.

### 3.5. BCYRN1 Knockdown Suppresses In Vivo HCC Tumor Growth

**Figure 6:**
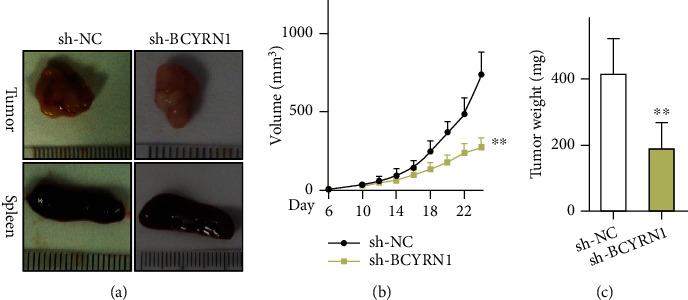
Suppressing BCYRN1 expression impairs HCC tumor progression. (a) Representative spleen and tumor images. (b) Tumor growth curves in the indicated groups. (c) Tumor weights at study end in the indicated groups. *n* = 7. ^∗^*P* < 0.05 vs. sh-NC.

To explore the in vivo relevance of BCYRN1 in a xenograft model of HCC, we next stably transfected HCC cells with lentiviruses encoding sh-NC or sh-BCYRN1 and monitored tumor formation following subcutaneous implantation in nude mice. Tumors were imaged at study end (Figure 6(a)), and tumor size and weight were additionally evaluated (Figures 6(b) and 6(c)). Relative to sh-NC tumors, those transduced with sh-BCYRN1 were significantly smaller and grew significantly more slowly. In line with our above results, BCYRN1 and BATF overexpression accelerated tumor growth, whereas TM4SF1 silencing had the opposite effect (Fig. S3). Together, these data suggest that BCYRN1 silencing can suppress the in vivo development of HCC.

## 4. Discussion

Recent work has highlighted the complex regulatory activities of different lncRNAs in the context of oncogenesis [13]. The dysregulation of lncRNAs has been associated with key cancer-related physiological and pathological cellular activities such as migration, proliferation, apoptosis, and invasion through a range of different molecular mechanisms [14]. These lncRNAs can thus serve as diagnostic, prognostic, or therapeutic biomarkers in specific cancer types [15]. Herein, we found that BCYRN1 was upregulated in HCC patient tumor tissues and that the degree of such upregulation was associated with decreased patient OS.

Brain cytoplasmic RNA 1 (BCYRN1, also known as BC200), encoded at chromosome 2p21, has emerged as a valuable prognostic biomarker in several solid tumors. Analyses of the 200 bp BCYRN1 RNA sequence have revealed three distinct sequence domains: a 5′*Alu* element, a central adenosine-rich region, and a 3′, 43-nucleotide, unique region containing a cytosine-rich stretch. From a functional perspective, BCYRN1 has previously been shown to influence tumor cell growth, apoptosis, and signal transduction through diverse complex pathways [16, 17]. In one prior report, this lncRNA was shown to sequester miR-619-5p and to thereby upregulate CUEDC2 expression and PTEN/AKT/p21 pathway activation, thus modulating glioma cell malignancy [18]. In this report, BCYRN1 knockdown was shown to suppress HCC proliferative and invasive activity in vitro and tumor growth in vivo. Similarly, researchers studying AGS cells have shown that the knockdown of this lncRNA can result in G1/G0 cell cycle arrest, enhanced apoptotic cell death, and disrupted cell migration [19]. BCYRN1 knockdown in cervical cancer has also been shown to disrupt tumor growth, reduce MMP3 and VEGF expression, and enhance miR-138 expression [20].

**Figure 7:**
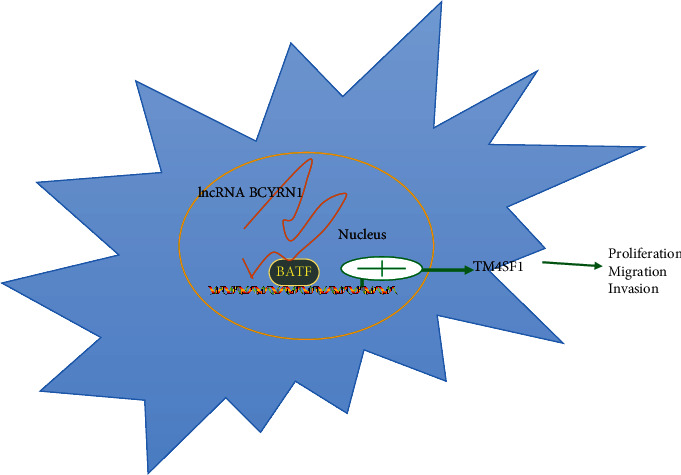
A schematic overview of the mechanisms whereby BCYRN1 modulates HCC progression. The lncRNA BCYRN1 recruits BATF to the TM4SF1 promoter, thereby driving its upregulation and enhancing malignant HCC cell proliferation, invasion, and migration.

The BATF transcription factor family is a group of AP-1 transcription factors including BATF, BATF2, and BATF3 [21]. BATF is encoded on chromosome 14q24.3 in humans and gives rise to a single transcript (NM_006399) [22], which encodes a nuclear alkaline leucine zipper protein. BATF negatively regulates AP-1/ATF transcriptional activity [23], thereby impacting cellular survival, proliferation, and differentiation [24]. Several studies have shown BATF to be capable of influencing oncogenesis [25, 26]. In line with such findings, we observed high levels of BATF expression in HCC tissues and cells in this study, wherein we found it to promote TM4SF1 upregulation. The overexpression of TM4SF1 was negatively correlated with patient survival, and BCYRN1 was able to influence the invasion, migration, and proliferation of HCC cells by recruiting BATF to upregulate TM4SF1 (Figure 7). TM4SF1 represents a potentially viable therapeutic target for the treatment of several cancers owing to its role in the context of tumor growth and progression [27, 28]. Overall, our data show that BCYRN1 can enhance TM4SF1 expression in a BATF recruitment-dependent fashion to modulate tumor malignancy, thereby enhancing HCC cell migration, invasion, and proliferation.

## 5. Conclusion

These results suggest that the BCYRN1/BATF/TM4SF1 axis tightly regulates HCC malignancy, making it an attractive target for therapeutic intervention. Overexpressing BCYRN1 can enhance HCC progression by driving BATF-mediated TM4SF1 upregulation. Future studies, however, will be necessary to validate and expand upon our data, and more work is required to understand the broader regulatory mechanisms active in this oncogenic context.

## Data Availability

The datasets used and/or analyzed during the current study are available from the corresponding author on reasonable request.

## Supplementary Materials

Supplementary 1 Supplementary Table 1. Sequences of primers.

Supplementary 2 Figure S1. The expression of BCYRN1 in HCC. (A) The lncMAP database was used to predict BCYRN1 expression profiles. (B) UALCAN datasets were scanned using the TCGA database to assess BATF expression. (C) The relationship between BATF expression and HCC prognosis was assessed via the UALCAN analysis. (D) mRNA level expression of TM4SF1 in HCC patient primary tissues from TCGA database was assessed with UALCAN. (E) UALCAN was used to gauge the association between TM4SF1 and HCC patient prognosis. ^∗^*P* < 0.05. Figure S2. BCYRN1 controls TM4SF1 expression to modulate the proliferation, invasion, and migration of HCC cells. (A) Proliferation was examined via colony formation assay. (B) HCC cell migration and invasion were evaluated in a Transwell assay. (C) BATF, TM4SF1, E-cadherin, and MMP2 levels in HCC cells in the OV-NC + sh-NC, OV-BCYRN1 + OV-BATF + sh-NC, or OV-BCYRN1 + OV-BATF + shTM4SF1 groups were assessed by Western blotting, with GAPDH for normalization. (D) Immunofluorescent staining of E-cadherin levels. Data are means ± SD from triplicate experiments. Figure S3. The BCYRN1/BATF/TM4SF1 axis controls in vivo HCC tumor growth. (A) Representative spleen and tumor images. (B) Xenograft tumor volumes were quantified over time. (C) Tumor weight was quantified. *n* = 7 mice/group; data are means ± SD and were compared using repeated-measures ANOVAs where appropriate.
