# Supplementary material for: Long Noncoding RNA BCYRN1 Recruits BATF to Promote TM4SF1 Upregulation and Enhance HCC Cell Proliferation and Invasion
Source: Dis Markers. 2022 Jun 11;2022:1561607. doi: 10.1155/2022/1561607 (PMC9206761; doi:10.1155/2022/1561607)
Supplement: Supplementary 1 — Supplementary Table 1. Sequences of primers. [file 1561607.f1.docx]

| Table S1: Sequences of primers | |
| --- | --- |
| Name | Sequences (5′-3′) |
| BCYRN1 Forward | GCCTGTAATCCCAGCTCTCA |
| BCYRN1 Reverse | GGTTGTTGCTTTGAGGGAAG |
| BATF Forward | TATTGCCGCCCAGAAGAGC |
| BATF Reverse | GCTTGATCTCCTTGCGTAGAG |
| TM4SF1 Forward | CGGCCAGTGGAACTACACC |
| TM4SF1 Reverse | GCCTCCAAGCACTCCATTTAT |
| GAPDH Forward | GGCATGGACTGTGGTCATGAG |
| GAPDH Reverse | CATGGGTGTGAACCATGAGAA |
| TM4SF1-P1-Forward | GGGGTACCGTTTCACCATGTTGGCCAGGATG |
| TM4SF1-P1-Reverse | CGACGCGTCAGACGAAATCCTTGGGACAGGC |
| TM4SF1-P2-Reverse | CGACGCGTGTGGTTACCTTGCAGAAGGAGGGG |
| TM4SF1-P3-Reverse | CGACGCGTTGAGTTCTCATGAGATCTGACAGT |
| TM4SF1-MUT1 Forward | GGGGAAACTGCTGCCACCTCCCCCCAGTCTCTCACT |
| TM4SF1-MUT1- Reverse | AGTGAGAGACTGGGGGGAGGTGGCAGCAGTTTCCCC |
| TM4SF1-MUT2 Forward | CACCTGAGCCCTTTTGCCACCTCTGTTGT |
| TM4SF1-MUT2- Reverse | ACAACAGAGGTGGCAAAAGGGCTCAGGTG |
