# Supplementary material for: Long Noncoding RNA BCYRN1 Recruits BATF to Promote TM4SF1 Upregulation and Enhance HCC Cell Proliferation and Invasion
Source: Dis Markers. 2022 Jun 11;2022:1561607. doi: 10.1155/2022/1561607 (PMC9206761; doi:10.1155/2022/1561607)
Supplement: Supplementary 2 — Figure S1. The expression of BCYRN1 in HCC. (A) The lncMAP database was used to predict BCYRN1 expression profiles. (B) UALCAN datasets were scanned using the TCGA database to assess BATF expression. (C) The relationship between BATF expression and HCC prognosis was assessed via the UALCAN analysis. (D) mRNA level expression of TM4SF1 in HCC patient primary tissues from TCGA database was assessed with UALCAN. (E) UALCAN was used to gauge the association between TM4SF1 and HCC patient prognosis. ∗P < 0.05. Figure S2. BCYRN1 controls TM4SF1 expression to modulate the proliferation, invasion, and migration of HCC cells. (A) Proliferation was examined via colony formation assay. (B) HCC cell migration and invasion were evaluated in a Transwell assay. (C) BATF, TM4SF1, E-cadherin, and MMP2 levels in HCC cells in the OV-NC + sh-NC, OV-BCYRN1 + OV-BATF + sh-NC, or OV-BCYRN1 + OV-BATF + shTM4SF1 groups were assessed by Western blotting, with GAPDH for normalization. (D) Immunofluorescent staining of E-cadherin levels. Data are means ± SD from triplicate experiments. Figure S3. The BCYRN1/BATF/TM4SF1 axis controls in vivo HCC tumor growth. (A) Representative spleen and tumor images. (B) Xenograft tumor volumes were quantified over time. (C) Tumor weight was quantified. n = 7 mice/group; data are means ± SD and were compared using repeated-measures ANOVAs where appropriate. [file 1561607.f2.docx]

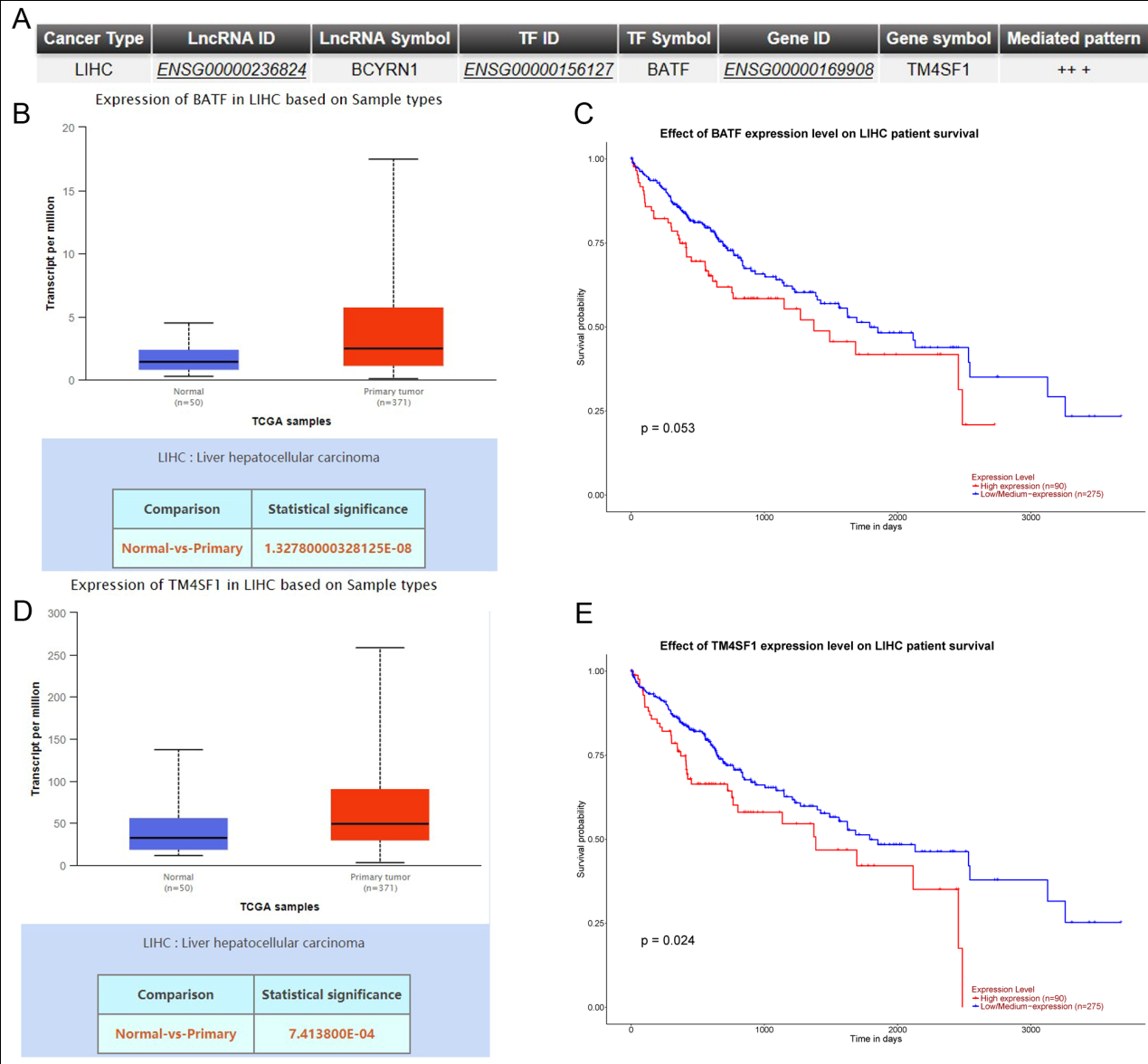


**Fig. S1**

The expression of BCYRN1 in HCC. (A) The LncMAP database was used to predict BCYRN1 expression profiles. (B) UALCAN datasets were scanned using the TCGA database to assess BATF expression. (C) The relationship between BATF expression and HCC prognosis was assessed via UALCAN analysis. (D) mRNA level expression of TM4SF1 in HCC patient primary tissues from the TCGA database was assessed with UALCAN. (E) UALCAN was used to gauge the association between TM4SF1 and HCC patient prognosis. * *P* <0.05.


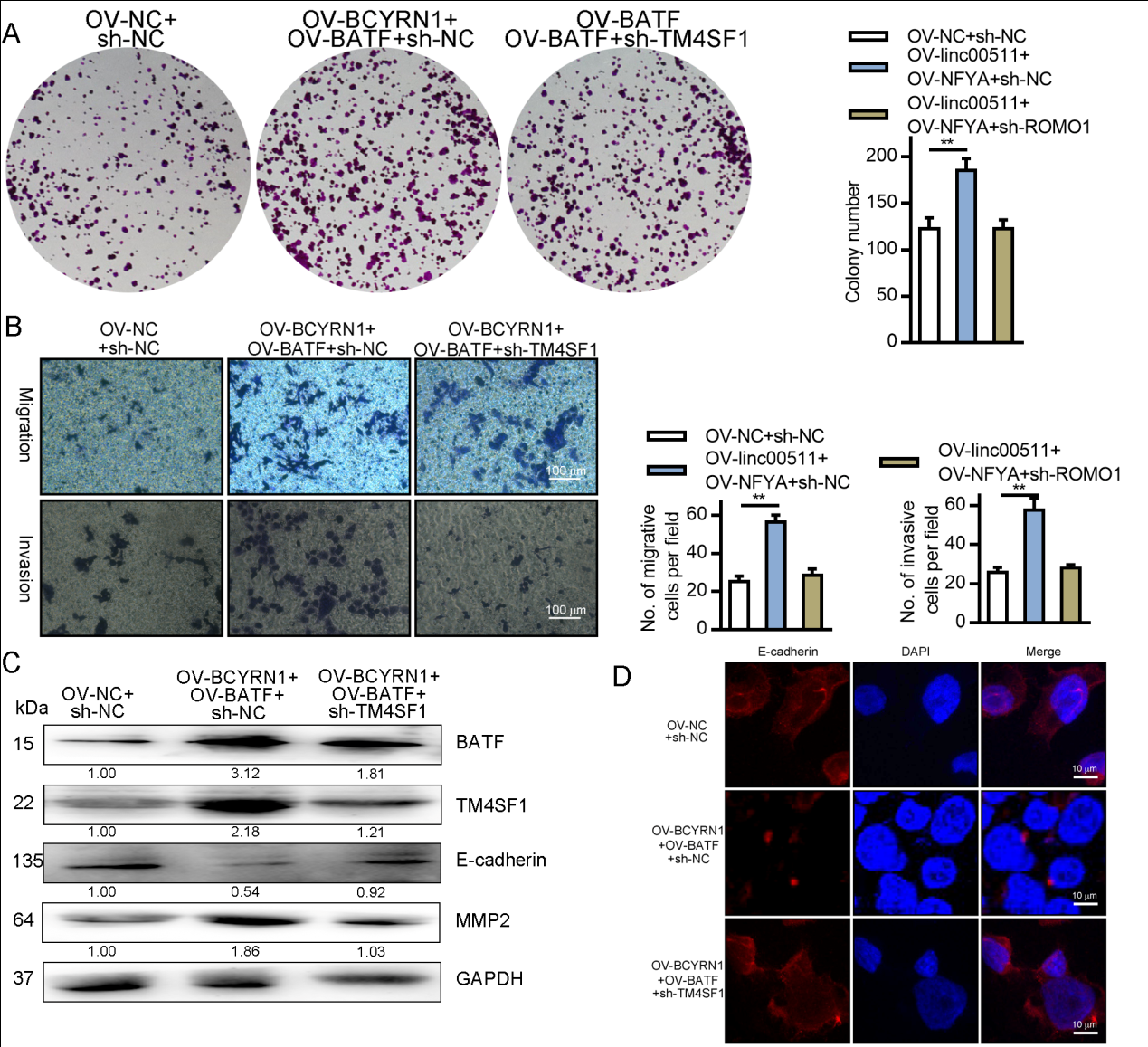


**Fig. S2**

BCYRN1 controls TM4SF1 expression to modulate the proliferation, invasion, and migration of HCC cells. (A) Proliferation was examined via colony formation assay. (B) HCC cell migration and invasion were evaluated in a Transwell assay. (C) BATF, TM4SF1, E-Cadherin, and MMP2 levels in HCC cells in the OV-NC + sh-NC, OV-BCYRN1 + OV-BATF + sh-NC, or OV-BCYRN1+OV-BATF+shTM4SF1 groups were assessed by Western blotting, with GAPDH for normalization. (D) Immunofluorescent staining of E-cadherin levels. Data are means ± SD from triplicate experiments.


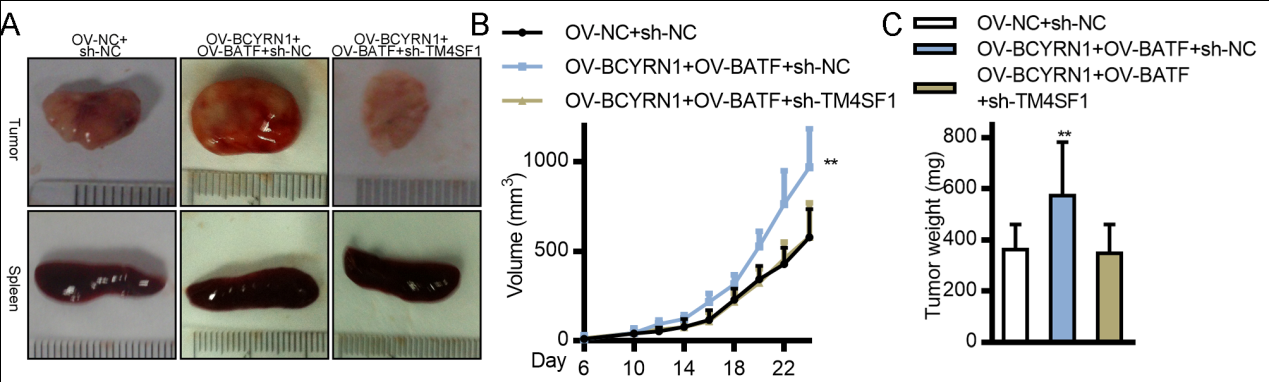


**Fig. S3**

The BCYRN1/BATF/TM4SF1 axis controls *in vivo* HCC tumor growth. (A) Representative spleen and tumor images. (B) Xenograft tumor volumes were quantified over time. (C) Tumor weight was quantified. n=7 mice/group; data are means ± SD and were compared using repeated-measures ANOVAs where appropriate.
